# Exploring Drug Treatment Patterns Based on the Action of Drug and Multilayer Network Model

**DOI:** 10.3390/ijms21145014

**Published:** 2020-07-16

**Authors:** Liang Yu, Yayong Shi, Quan Zou, Shuhang Wang, Liping Zheng, Lin Gao

**Affiliations:** 1 School of Computer Science and Technology, Xidian University, Xi’an 710071, China; 15229288213@163.com (Y.S.); lgao@mail.xidian.edu.cn (L.G.); 2 Institute of Fundamental and Frontier Sciences, University of Electronic Science and Technology, Chengdu 650004, China; zouquan@nclab.net; 3 Department of Radiology, Massachusetts General Hospital, Boston, MA 02114, USA; swang38@mgh.harvard.edu; 4 School of Computer Science and Technology, Liaocheng University, Liaocheng 252000, China; zhengliping80@163.com

**Keywords:** drug treatment pattern, drug-target module, multilayer network, tissue specificity, drug action

## Abstract

Some drugs can be used to treat multiple diseases, suggesting potential patterns in drug treatment. Determination of drug treatment patterns can improve our understanding of the mechanisms of drug action, enabling drug repurposing. A drug can be associated with a multilayer tissue-specific protein–protein interaction (TSPPI) network for the diseases it is used to treat. Proteins usually interact with other proteins to achieve functions that cause diseases. Hence, studying drug treatment patterns is similar to studying common module structures in multilayer TSPPI networks. Therefore, we propose a network-based model to study the treatment patterns of drugs. The method was designated SDTP (studying drug treatment pattern) and was based on drug effects and a multilayer network model. To demonstrate the application of the SDTP method, we focused on analysis of trichostatin A (TSA) in leukemia, breast cancer, and prostate cancer. We constructed a TSPPI multilayer network and obtained candidate drug-target modules from the network. Gene ontology analysis provided insights into the significance of the drug-target modules and co-expression networks. Finally, two modules were obtained as potential treatment patterns for TSA. Through analysis of the significance, composition, and functions of the selected drug-target modules, we validated the feasibility and rationality of our proposed SDTP method for identifying drug treatment patterns. In summary, our novel approach used a multilayer network model to overcome the shortcomings of single-layer networks and combined the network with information on drug activity. Based on the discovered drug treatment patterns, we can predict the potential diseases that the drug can treat. That is, if a disease-related protein module has a similar structure, then the drug is likely to be a potential drug for the treatment of the disease.

## 1. Introduction

Drugs interact with target and non-target molecules, thus triggering downstream signal cascades and disrupting the cell’s transcriptome [1]. Discovering new drug targets is critical for improving our understanding of the mechanisms of drug action [2], which is essential for drug discovery [3,4,5,6], clinical trials, and overcoming drug resistance [7,8]. The mechanisms mediating the effects of drugs on targets have been widely studied [9,10,11,12,13,14,15]. Drug targets are not only limited to a single gene and can also include modules or pathways that participate in the regulation of disease processes [16]. Although each gene in the drug-target module may not be helpful for disease treatment, combinations of the genes within the module may play important roles in disease treatment [16]. By targeting multiple genes in the drug-target module, it may be possible to identify the function of genes related to complex disease pathology at the tissue level [17,18].

Many drugs have been shown to be effective against a variety of diseases [19], indicating that drug treatment may exhibit a certain pattern. Genes in drug-target modules are highly expressed in disease-associated tissues [20,21,22], allowing drug targets to be treated by specific small-molecule drugs [23,24,25,26]. In a previous study [27], researchers assumed that each disease corresponds to a tissue-specific protein–protein interaction (TSPPI) network, suggesting that a single drug could be associated with a multilayer TSPPI network. Because biological networks complement each other [28,29], many studies in this field have focused on multilayer networks [30]. Accordingly, it may be helpful to explore drug treatment patterns by extracting target modules from multilayer TSPPI networks.

**Figure 1:**
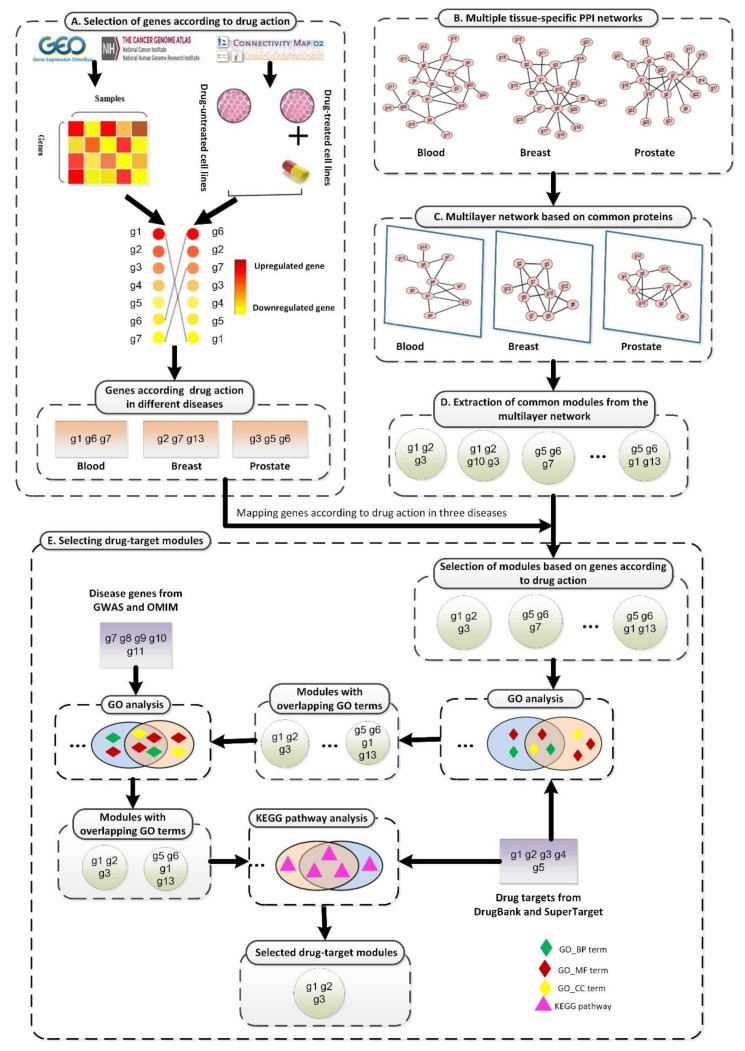
The framework of the proposed SDTP method. (**A**) Selection of genes according to TSA activity in three diseases based on gene differential expression profiles. (**B**) Processing of TSPPI networks related to the three diseases from the GIANT database. (**C**) Standardization of a multilayer subgraph based on common proteins obtained from (**B**). (**D**) Use of the tensor-based mining algorithm to identify drug-target modules from the multilayer TSPPI network. (**E**) Selection and validation of drug-target modules.

In this report, we propose a new method to study drug treatment patterns based on drug activity and a multilayer network model, as shown in Figure 1. First, gene expression data obtained from the Gene Expression Omnibus (GEO) [31], The Cancer Genome Atlas (TCGA) [32], and the Connectivity Map (CMAP) [33,34] were preprocessed (Figure 1A). Based on the expression values of genes in disease states and drugs, we selected genes that played a key role when drugs acted on each disease. Next, we preprocessed multiple TSPPI networks downloaded from the Genome-scale Integrated Analysis of Gene Networks in Tissues (GIANT) database (Figure 1B) [27]. We selected edges with higher weights to improve the reliability of TSPPI networks. We then selected common genes from multiple high-quality TSPPI networks (Figure 1C) and created relevant subgraphs for each TSPPI network. These subgraphs were then normalized to form a multilayer network with the same nodes and no interaction between layers, and a multilayer network module mining algorithm was used to identify candidate drug target modules (Figure 1D). Finally, we performed a series of filtering processes on all candidate drug target modules and used the remaining modules as potential drug targets (Figure 1E). We performed a case study using the drug trichostatin A (TSA) [35] and three diseases (leukemia, breast cancer, and prostate cancer) that are commonly treated with TSA [36,37,38,39,40]. Our analysis identified two drug-target modules for TSA (M17 and M18) as potential treatment patterns for TSA.

Key points:

- For the first time, we proposed a network-based model to analyze the drug treatment patterns.
- The new framework to study the treatment pattern of drugs was based on the action of drug and multilayer network model.
- Taking drug TSA as a case, we found two modules from a tissue-specific multilayer protein-protein network as TSA’s treatment patterns.
- By analyzing the significance, composition, and functions, the two modules were proven to be the potential treatment patterns of TSA.
- Analysis of the treatment patterns of the drug through the network method provides novel solutions for disease treatment.

## 2. Materials and Methods

To develop and validate our proposed method, our study case used TSA and three diseases that are commonly treated with TSA (leukemia, breast cancer, and prostate cancer).

### 2.1. Datasets

#### 2.1.1. Gene Expression Data for TSA Activity

From the CMAP database [33,34], we downloaded gene expression data for multiple samples related to TSA activity. The gene expression data represent the gene expression values before and after using TSA on cell lines of different diseases. The data were obtained from MCF7, PC3, and HL60 cells using HT_HG-U133A chips.

#### 2.1.2. Gene Expression Data According to Disease State

We downloaded gene expression profiles for breast cancer and prostate cancer from TCGA database [41]. This gene expression profile represent the gene expression values of a patient in a disease state. The breast cancer data were obtained from 1212 samples (1100 tumor samples and 112 control samples). The prostate cancer data were obtained from 550 samples (498 tumor samples and 52 control samples). We downloaded the gene expression profiles for leukemia from the NCBI GEO database [31]. The gene expression data (GSE48558) were obtained from 170 samples (121 tumor samples and 49 control samples).

#### 2.1.3. TSPPI Networks

We downloaded weighted TSPPI networks for breast cancer, prostate cancer, and blood tissues marked as “top edges” from the GIANT database [27]. The TSPPI network represents the interaction network between genes in the corresponding tissue. “Top edges” signified that the network was filtered to include only edges that had evidence (weight) supporting a tissue-specific functional interaction.

### 2.2. Standardizing Networks

We used the following equation to standardize the weights of three TSPPI networks (breast, prostate, and blood tissues):

(1) $$X_{after}=\frac{\left(X_{after\_max}-X_{after\_min})*(X_{before}-X_{before\_min}\right)}{\left(X_{before\_max}-X_{before\_min}\right)}+X_{after\_min}$$

where $X_{after\_max}$ and $X_{after\_min}$ were set to 1 and 0.1, respectively.$X_{before\_max}$ and $X_{before\_min}$ represented the maximum and minimum weights of edges before the normalization, respectively. $X_{before}$ and $X_{after}$ represented the weights of edges before and after normalization, respectively. Further, we processed the three normalized networks based on nodes, edges, and node degree distribution information.

### 2.3. Selecting Differentially Expressed Genes

For gene expression profiles of the disease conditions (leukemia, breast cancer, and prostate cancer) and TSA activity, we used the Limma package [42] to analyze the differential expression of genes in cases and controls. The logFC value was used to evaluate the differential expression of genes. If $\log FC_{i}>0$, gene *i* was an upregulated gene; if $\log FC_{i}=0$, gene *i* did not differ between cases and controls, and if $\log FC_{i}<0$, gene *i* was a downregulated gene.

Finally, we obtained three sets of differentially expressed genes ($G_{l}$, $G_{b}$, and $G_{p}$) in three different disease states (l = leukemia, b = breast cancer, and p = prostate cancer) and a set of differentially expressed genes $G_{TSA}$ related to TSA activity. For example, if a gene in $G_{l}$ was upregulated or downregulated in $G_{TSA}$, then this gene was selected. Accordingly, we selected a subset of genes from $G_{l}$ that were critical for the treatment effects of TSA in leukemia. Finally, we obtained 824, 1213, and 1160 genes for leukemia, breast cancer, and prostate cancer, respectively.

### 2.4. Mining Modules from the Multilayer Network

In this study, we used a tensor-based computational framework to mine recurrent heavy subgraphs (RHSs) in multilayer networks, as proposed by Li et al. [43]. For any given m networks with the same n nodes that have different topologies, the following third-order tensor A could be used to represent the network [43]:

(2) $$A={\left(a_{ijk}\right)}_{n\times n\times m}$$

where $a_{ijk}$ indicates the weight of the edge between vertices *i* and j in the $k^{th}$ network. If i=j, $a_{ijk}=0$. If the network is a unidirectional network, $a_{ijk}=a_{jik}$. Candidate drug-target modules in disease multilayer networks based on tensor recognition can be identified by heaviness [43]. The heaviness of an RHS is defined as the summed weight of all edges in the RHS [43]:

(3) $$H_{A}(x,y)=\frac{1}{2}\sum_{i=1}^{n}\sum_{j=1}^{n}\sum_{k=1}^{m}a_{ijk}x_{i}x_{j}y_{k}$$

The gene vector $x={\left(x_{1},\ldots ,x_{n}\right)}^{T}$, where $x_{i}=1$ if gene *i* belongs to the RHS, and $x_{i}=0$ otherwise. The network vector $y={\left(y_{1},\ldots ,y_{n}\right)}^{T}$, where $y_{j}=1$ if the RHS appears in network j, and $y_{j}=0$ otherwise. In this study, we used RHSs with high heaviness as candidate drug-target modules for TSA.

### 2.5. Quantifying the Overlap between Modules

Modules containing at least three nodes were selected. We used the following measure to quantify the overlapping coefficient *c* between two modules:

(4) $$c=\frac{\left|A\cap B\right|}{\min(|A|,|B|)}$$

where *A* and *B* were the set of genes in two modules, respectively, |A∩B| was the number of elements in the intersection of sets *A* and *B*, and min(|A|,|B|) was the minimum value of the number of elements in *A* and *B*. A higher c value indicated that the two modules were similar. If $c\geq \frac{2}{3}$, the two modules were defined as overlapped modules.

## 3. Results

### 3.1. Constructing Three-Layer Tissue-Specific Networks

#### 3.1.1. Nodes and Edges

**Figure 2:**
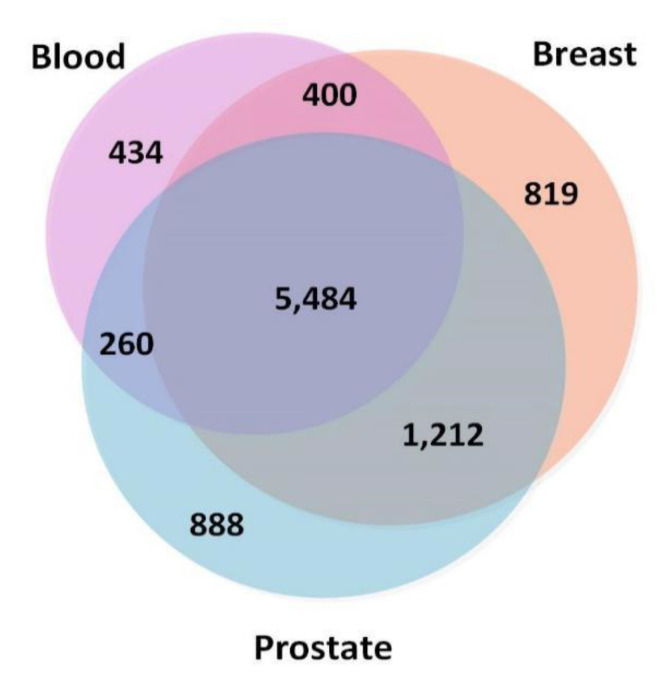
Numbers of overlapping genes in three tissue-specific networks (blood, breast, and prostate).

To ensure that the TSPPI networks had a similar density, we selected 173,072 edges (top 0.5%), 170,017 edges (top 0.25%), and 167,211 edges (top 0.25%) from the blood, breast, and prostate networks, respectively. The node information is shown in Figure 2. There were 5484 common nodes. From Figure 2, we found that most genes were involved in various cancers and only a small portion of the genes were tissue-specific [44].

#### 3.1.2. Degree Distribution

**Figure 3:**
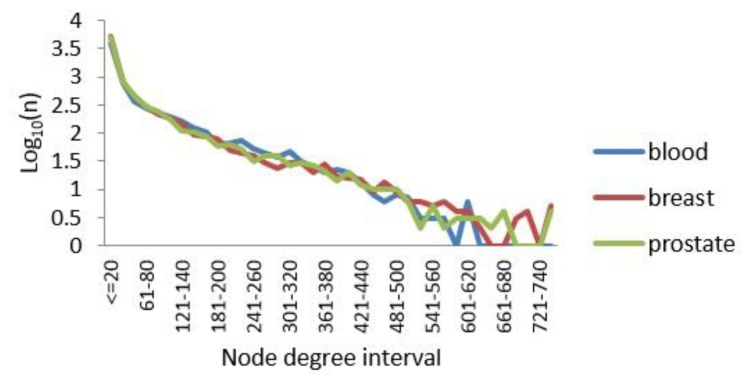
Node degree distributions for the three TSPPI networks. The X axis represents the distribution interval of the node degree. The Y axis represents Log_10_(n), where *n* is the number of nodes.

PPI networks are subject to the general distribution of scale-free networks [45], with only a few nodes having a large degree and most nodes having a relatively small degree of distribution. Here, the degree of a node in a network was set as the number of connections the node has with other nodes. We calculated the degrees of all nodes while drawing their degree distributions. The results are shown in Figure 3. Notably, a small number of nodes had a degree greater than 500, and these nodes were called hub nodes. The data also showed that our processing of the edges of the three TSPPI networks was reasonable. Therefore, in the following sections, we analyzed the three-layer TSPPI networks with filtered edges.

### 3.2. Selecting Parameter Heaviness

**Figure 4:**
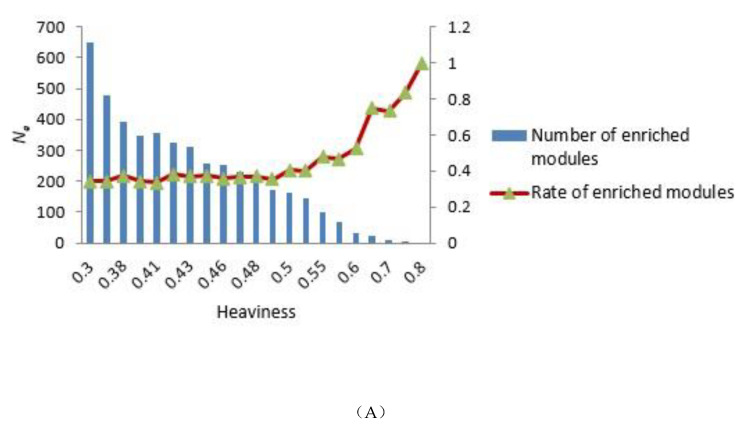

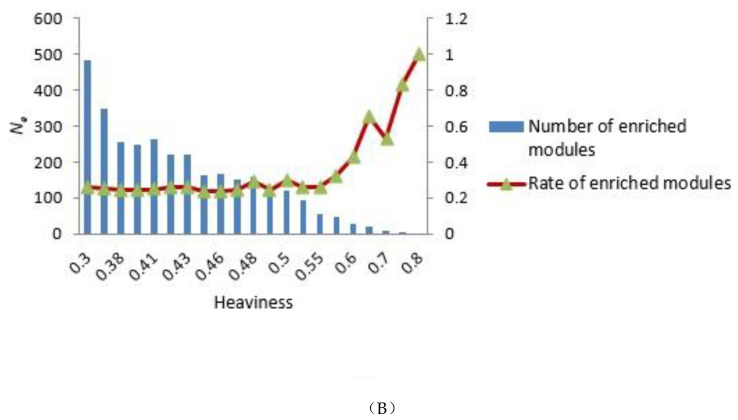
Distribution of the number of modules that could be significantly enriched in GO terms and KEGG pathways. (**A**,**B**) GO terms and KEGG pathways analyses, respectively. The blue bars represent the number of modules that were significantly enriched in GO terms (**A**) and KEGG pathways (**B**) under different heaviness values, i.e., Ne. The Y coordinate on the left corresponds to changes in Ne. The red curve with triangles indicates the ratio Rea between Ne and the total number of modules Na. The Y coordinate on the right corresponds to the value of Rea.

To construct the tensor-based computational framework for mining RHSs in multilayer networks, one challenge is the determination of the key parameter value called heaviness [43] (see Formula (3)). In order to obtain the appropriate heaviness value, we determined the number of modules that could be significantly enriched in gene ontology (GO) terms and Kyoto Encyclopedia of Genes and Genomes (KEGG) pathways under different heaviness values, as shown in Figure 4A,B. As heaviness grew, the number of enriched modules Ne in GO data and KEGG pathways continued to decrease, whereas the ratio $R_{ea}=N_{e}/N_{a}$ (Na is the total number of modules) increased. When heaviness was near 0.41, Rea changed smoothly, and when heaviness was equal to 0.41, Ne was slightly higher than its neighbor area.

**Figure 5:**
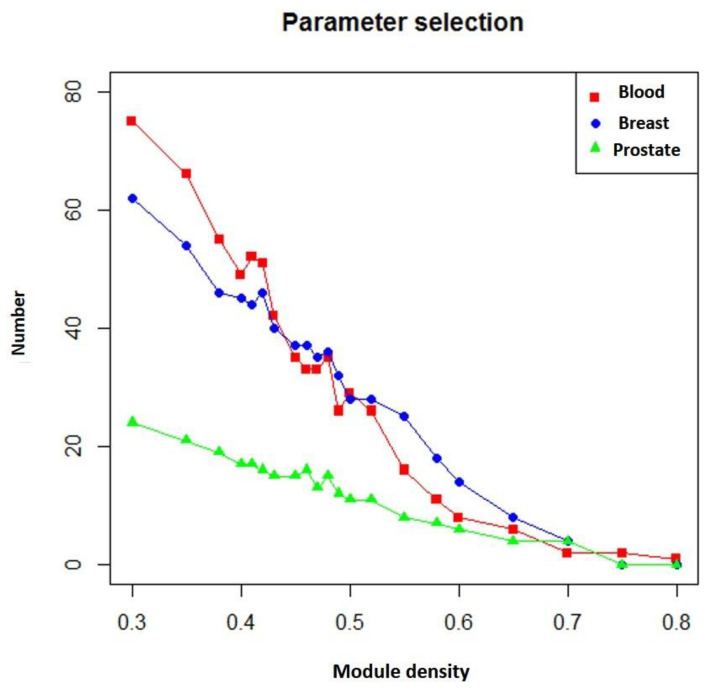
Numbers of overlapping genes between modules generated under different heaviness values and genes affected by TSA in different diseases. Red line: blood (leukemia); blue line: breast (breast cancer); green line: prostate (prostate cancer). The X axis represents heaviness values (“module density), and the Y axis indicates the number of overlapping genes (“number”).

In addition, if more known targets of the drug appeared in the module, the module was more likely to be a potential module target. Figure 5 shows the number of overlapping genes between the extracted modules and genes that TSA affected in three different diseases. We found that as module density (heaviness) increased, the number of overlapping genes decreased. Moreover, when compared with neighboring parameters, when module density (heaviness) was 0.41, the number of overlapping genes for leukemia (blood tissue) tended to increase (red line in Figure 5). For prostate cancer, a similar phenomenon was observed (green line in Figure 5). Therefore, we chose 0.41 as the value of heaviness. The drug-target modules obtained under this value were then analyzed.

### 3.3. Comparison of Predicted Modules between Three-Layer and Single-Layer Networks

#### 3.3.1. Comparison of Overlap between Modules

**Figure 6:**
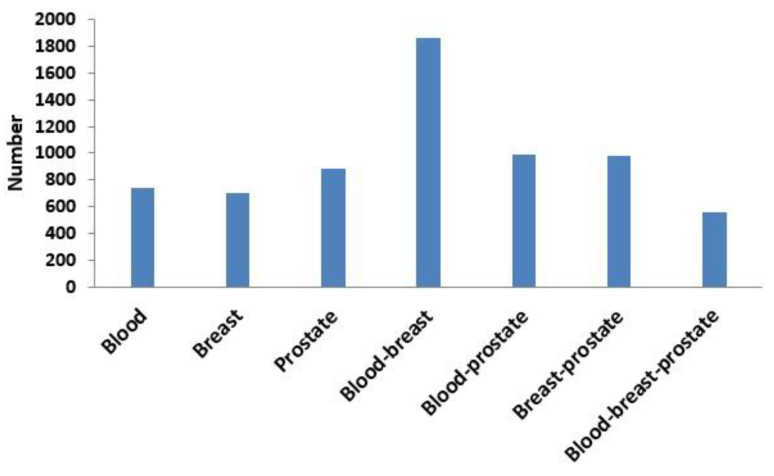
Numbers of overlapping modules between the single-layer network (blood, breast, or prostate) and three-layer network and between two or three single-layer networks.

**Figure 7:**
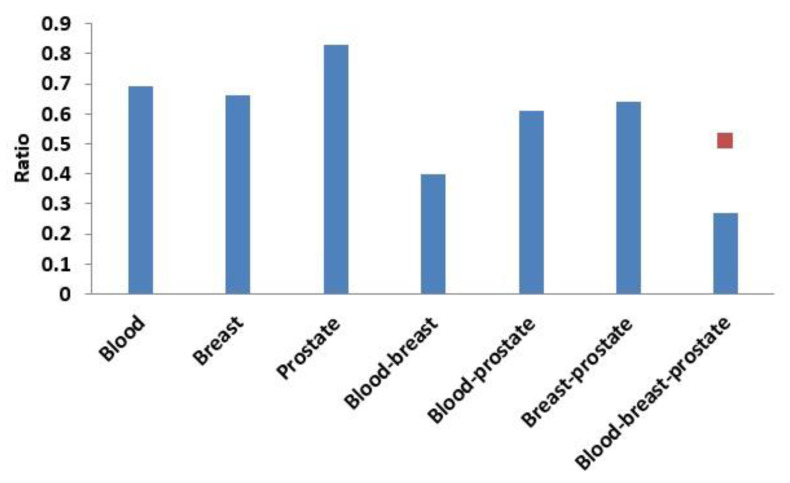
Ratio of the number of overlapping modules and the number of modules obtained from the three-layer network. The red squares represent proportions of the numbers of overlapping modules between the blood-breast-prostate and three-layer network modules to the number of blood-breast-prostate modules.

Next, based on the same module mining algorithm, we compared the overlap between modules mined in a single-layer network and modules mined in the three-layer network. The definition of overlap between two modules is shown in Equation (4), and the results of the comparisons are shown in Figure 6 and Figure 7. In Figure 6, if X was blood (or breast or prostate), its corresponding Y represented the number of overlapping modules between the single-layer blood (or breast or prostate) network and the three-layer network. If X was a combination of multiple single-layer networks, such as X = blood-breast, its corresponding Y represented the number of overlapping modules between the blood and breast PPI networks. Figure 7 shows the ratios between the numbers of overlapping modules shown in Figure 6 and the numbers of modules obtained from the multilayer network. The results from Figure 6 and Figure 7 showed that there were 285 (560 × 0.51 = 285) overlapping modules between the blood–breast–prostate modules and the three-layer network modules. Through this comparison, we found that modules in most single-layer networks could be detected and new modules could be found based on the multilayer network mining method. Information can be complementary between multilayer networks [46]. Therefore, the use of multilayer network mining algorithms could yield more meaningful modules [43,47].

#### 3.3.2. Functional Enrichment Comparison

**Figure 8:**
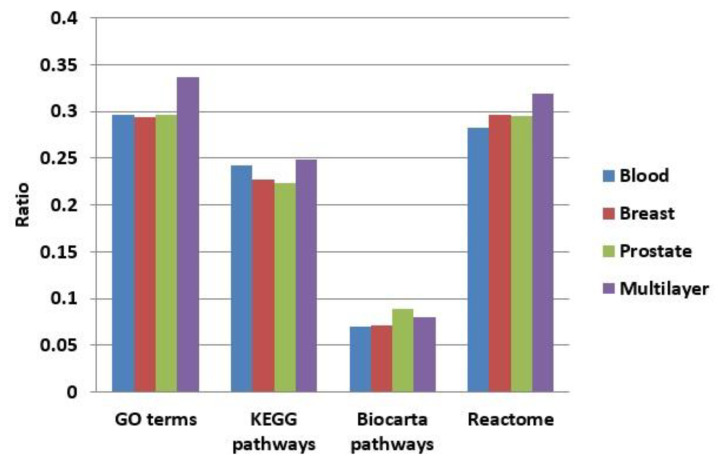
Comparison of the proportions of functionally enriched modules obtained for different networks using four enrichment methods.

We enriched the functions of modules in our multilayer and three single-layer networks (blood, breast, and prostate) using GO terms [48], KEGG pathways [49], BioCarta, and Reactome. Figure 8 shows a comparison of the proportions of functionally enriched modules obtained in different networks. Except for BioCarta pathways, the enrichment proportions of multilayer modules were all higher than those of single-layer modules. For BioCarta, the enrichment proportion of multilayer modules, although not as high as that of the modules in the prostate network, was slightly higher than the other two (blood and breast) single-layer networks.

### 3.4. Filtering Extracted Modules in the Multilayer Network

In total, we found 1063 drug-target modules from the multilayer network. To improve the reliability of drug-target modules, we filtered them based on sequential three steps, i.e., TSA activity, GO terms, and KEGG pathways.

#### 3.4.1. Analysis Based on TSA Activity

If a drug-target module contained differentially expressed genes, it was selected for further analysis. Based on this principle, 26 modules were selected, as shown in Table 1. For convenience, we numbered these modules M1–M26.

#### 3.4.2. Analysis Based on GO Terms

Next, the 26 selected modules in Table 1 were subjected to additional processing. GO [48] is a framework for biological models that divides genes based on molecular functions (MFs), cellular components (CCs), and biological processes (BPs). A module was chosen if the module had overlapping GO terms with the TSA target genes. Furthermore, we also ensured that the module was related to the disease; thus, the module had overlapping GO terms with disease-causing genes. We further filtered the 26 selected modules using two steps.

**Figure 9:**
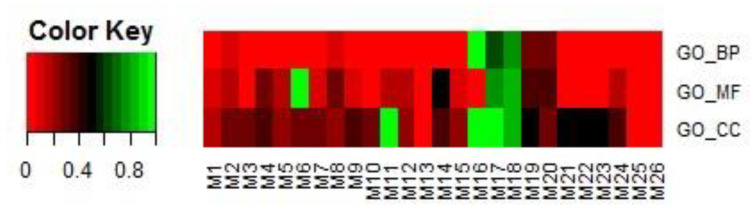
Proportions of the numbers of common terms between each of the 26 selected modules and TSA targets among the total number of terms related to the module. Terms included BPs, MFs, and CCs.

In the first step, modules were filtered by overlapping GO terms with TSA targets. We downloaded the targets of TSA through SuperTarget (http://insilico.charite.de/supertarget/), a database developed to collect information about drug-target relations [50], and DrugBank (https://www.drugbank.ca/), a unique bioinformatics and cheminformatics resource that combines detailed drug data with comprehensive drug-target information databases [51]. TSA targets were mapped to DAVID (version 6.8) [52,53] for GO enrichment analysis. In total, 74 GO terms were enriched, including 42 BP terms, 20 MF terms, and 12 CC terms. For each of the 26 modules, the same analysis in DAVID was performed. That is, each module corresponded to a GO term list, including BP, MF, and CC terms. For each of the BP, MF, and CC terms, we first obtained the number of terms common to each module and the corresponding TSA targets. We then calculated the proportion of the number of common terms among the total number of terms related to each module. The results are shown in Figure 9.

If a module had more overlapping GO terms in BPs, MFs, and CCs, it was selected in this step for further analysis in step 2. In this study, eight modules, i.e., M1, M2, M3, M8, M17, M18, M19, and M20, were selected.

**Figure 10:**
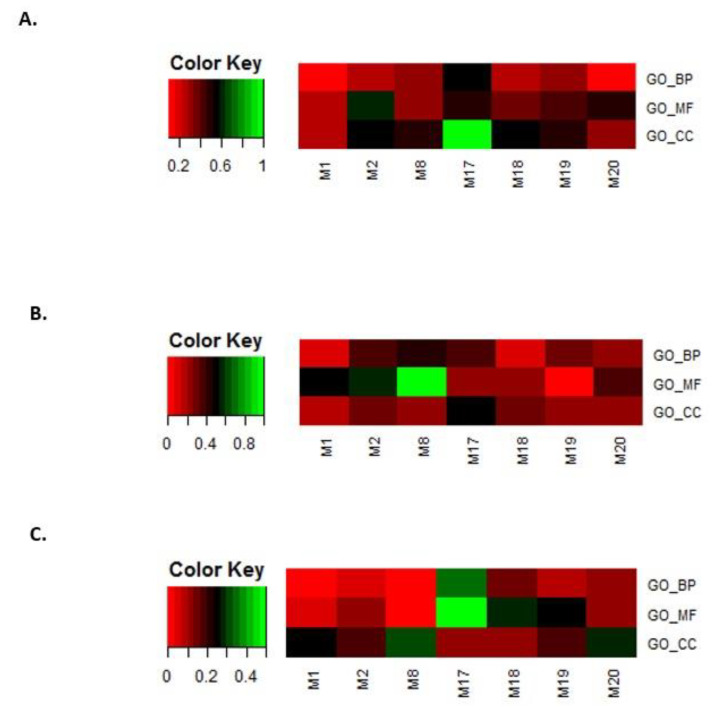
Overlap rates between disease-enriched GO terms and the eight selected module-enriched GO terms. (**A**) leukemia, (**B**) breast cancer, and (**C**) prostate cancer.

In the second step, the eight modules selected in step 1 were filtered by overlapping GO terms with disease genes. Genes related to leukemia, breast cancer, and prostate cancer were downloaded from OMIM [54,55] and GWAS databases [56]. The same GO enrichment analysis was performed. For leukemia, breast cancer, and prostate cancer, the overlap rates between the disease-enriched GO terms and the selected eight module-enriched GO terms are shown in Figure 10A–C.

We only kept modules that overlapped with the BP, MF, and CC terms that were enriched for the three disease gene sets. In this way, we obtained four modules, i.e., M2, M17, M18, and M20.

#### 3.4.3. Analysis Based on KEGG Pathways

To further elucidate the relationships between modules, we also analyzed the relationships between internal genes and their first-order neighbors in the modules. The external genes were more closely related to the module and were more affected by the module. The strength of the relationship between a module D and its first-order gene j was defined as:

(5) $$score(j,D)=\sum_{i\in D}w_{ij}$$

where $w_{ij}$ represents the weight between i(i∈D) and j.

**Figure 11:**
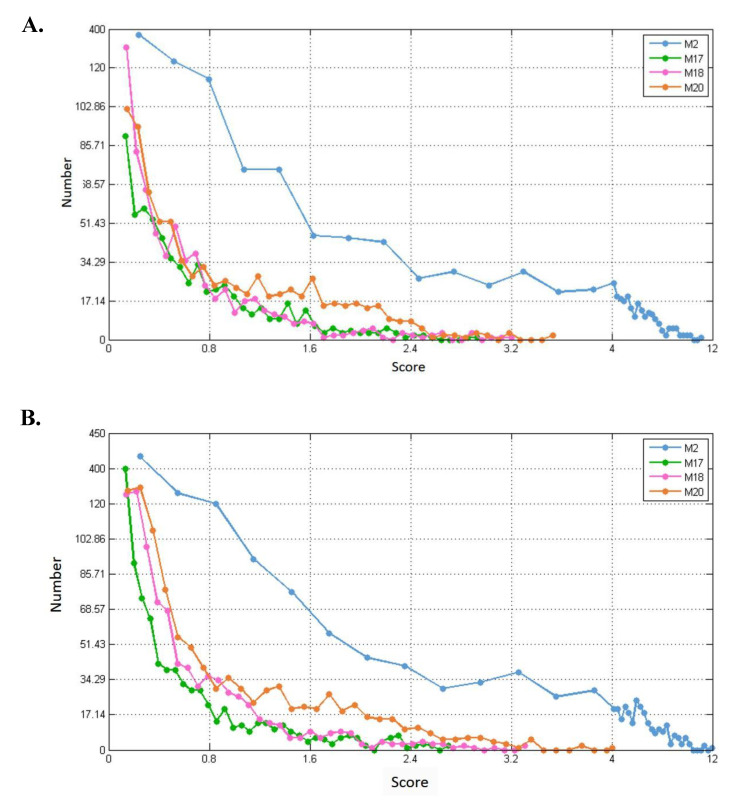

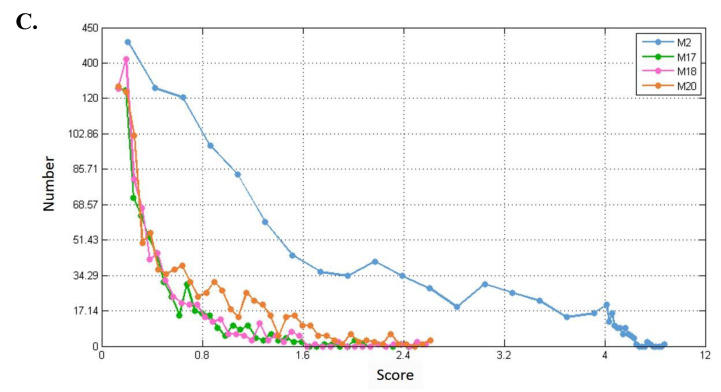
Distribution of connection strengths between four modules and their corresponding first-order genes in three tissue-specific networks. The X axis represents the connection strength. The Y axis indicates the number of first-order genes of modules. (**A**–**C**) show blood, breast, and prostate TSPPI networks, respectively.

For each of the modules (M2, M17, M18, and M20), we separately calculated their connection strength with their first-order nodes in blood, breast, and prostate TSPPI networks. The results are shown in Figure 11A–C. For each module, we selected first-order genes with high scores to analyze KEGG pathway enrichment by DAVID.

In different networks, the distribution of first-order neighbor nodes of the same module was similar. When there was a different number of nodes in each module, the number of first-order neighbors differed. Thus, the four modules had different first-order neighbors in the same network. We used the following method to filter the first-order neighbors for each module. First, we selected 1,000,000 genes randomly from the first-order genes of a module and determined their connection strength scores (see Formula (5)). We sorted all the scores in descending order and selected the 50th value as the threshold. Finally, genes whose scores were greater than this threshold were retained. For each of the four modules, we performed KEGG pathway enrichment analysis for the modules and their preserved first-order neighbors. The results for blood, breast, and prostate tissue-specific networks are shown in Table 2, Table 3 and Table 4. For example, in Table 2, for module M2, the threshold 7.0 was used to filter the first-order genes. Forty-six first-order genes were conserved and enriched in 12 KEGG pathways.

Drugs can treat the target diseases by affecting first-order neighbors of the module. The more the KEGG pathways overlapped between the first-order genes of the module and TSA targets, the better the therapeutic effect. Table 5 shows the number of KEGG pathways that overlapped between the first-order genes of the module and the TSA targets. Finally, two modules (M17 and M18) were selected.

In addition, we also analyzed the KEGG pathways that overlapped between the first-order genes of M17 and M18 and genes that caused leukemia, breast cancer, and prostate cancer. We found that overlap was enhanced. Table 6 shows the number of overlapping KEGG pathways. The results verified that TSA could be used to treat these three diseases and that the two identified modules, M17 and M18, were likely to indicate the TSA treatment pattern.

### 3.5. Validating and Analyzing the Significance of M17 and M18

#### 3.5.1. Statistical Significance

To verify that modules M17 and M18 were biologically significant, we performed the following five-step analysis using the blood, breast, and prostate TSPPI networks: (1) the sum of the weights of the inner edges of module m(m = M17 or M18) in the TSPPI network was calculated and saved as $S_{m}$; (2) the TSPPI network used in step 1 was randomly disturbed, the network connections were unchanged, and the weights of the edges were randomly disrupted; (3) in the randomized network obtained in step 2, the sum of the weights of the inner edges of module m was calculated again; (4) steps 2 and 3 were repeated 106 times, and the results were saved as $S_{m}^{\left(n\right)}\;(n=1,2,\ldots ,10^{6})$; and (5) the result $S_{m}^{\left(n\right)}$ obtained in step 4 was compared with the result $S_{m}$ obtained in step 1 using the following formula:

(6) $$p=\frac{\sum_{n=1}^{10^{6}}count(S_{m}\leq S_{m}^{\left(n\right)})}{10^{6}}$$

where p denotes the p value of the drug-target module m. If $S_{m}\leq S_{m}^{\left(n\right)}$, $count(S_{m}\leq S_{m}^{\left(n\right)})=1$; otherwise,$count(S_{m}\leq S_{m}^{\left(n\right)})=0$. Smaller p values were associated with more meaningful modules. Based on the above analysis, we obtained the p values of M17 and M18. The results are shown in Table 7.

From Table 7, we found that the p values of M17 and M18 were both less than 0.05. The results demonstrated that the target modules M17 and M18 for TSA were highly statistically significant and that their internal genes had strong interactions.

#### 3.5.2. Significance of Other TSA-Related TSPPI Networks

To further verify the close correlations of modules M17 and M18 with TSA, we also analyzed the performance of M17 and M18 in TSPPI networks related to other diseases treated with TSA. We obtained five other TSA-treated cancers marked as “T” in the CTD [32], i.e., lung cancer, colon cancer, ovarian cancer, pancreatic cancer, and myeloma. Their TSPPI networks were all downloaded from GIANT, and edges with low weights were deleted. The network preprocessing procedures were the same as those described in Section 3.1. For the five processed networks, we also calculated the *p* values of module targets M17 and M18. The results are shown in Table 8.

As shown in Table 8, the *p* values were all less than 0.05. These findings indicated the importance of M17 and M18 in these TSPPI networks, suggesting that TSA may be used to treat other diseases by acting on M17 and M18. Therefore, these results were strong evidence supporting M17 and M18 as a treatment pattern for TSA.

### 3.6. Differential Analysis of Internal Connections in M17 and M18 for Co-Expression Networks

**Figure 12:**
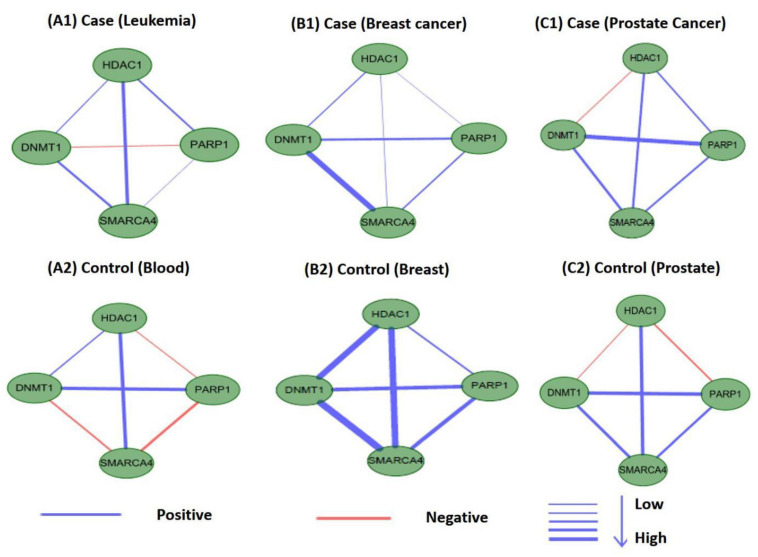
Differences in the co-expression network connections of M17 in normal and tumor-based states under three different conditions (leukemia, breast cancer, and prostate cancer).

**Figure 13:**
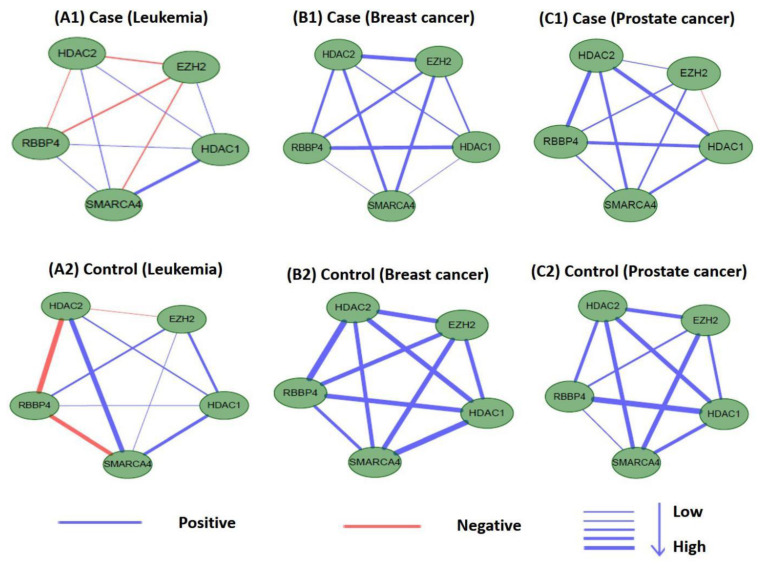
Differences in the co-expression network connections of M18 in normal and tumor-based states under three different conditions (leukemia, breast cancer, and prostate cancer).

Perturbations in network characteristics induced by genetic variation cause phenotypic changes, such as disease [57]. Next, we investigated the differential connections within modules of M17 and M18 in co-expression networks under disease and normal conditions. We used Pearson correlation coefficients to establish co-expression network connections for genes in M17 and M18 in disease and control samples. We found that the connections differed substantially. Figure 12 and Figure 13 showed differences in the co-expression network connections of M17 and M18. For example, in the control (normal) and case (tumor) samples for leukemia (Figure 12(A1,A2)), none of the gene pairs in M17 shared similar relationships in the two co-expression networks. For module M18, the connection differences between genes in case and control samples were very large, e.g., in leukemia (Figure 13(A1,A2)). These results indicated that leukemia, breast cancer, and prostate cancer were closely related to modules M17 and M18 and that these modules may cause different diseases owing to changes in connectivity under different conditions. However, these findings also suggested that the modules M17 and M18 were likely potential treatment patterns for TSA.

### 3.7. PubMed Literature Validation of Genes in Modules M17 and M18

M17 contained four genes: histone deacetylase 1 (*HDAC1*), poly-ADP ribose polymerase 1 (*PARP1*), DNA methyltransferase 1 (*DNMT1*), and *SWI/SNF* related, matrix associated, actin dependent regulator of chromatin, subfamily A, member 4 (*SMARCA4*), whereas M18 contained five genes: *HDAC2*, *HDAC1*, RB binding protein 4 (*RBBP4*), enhancer of Zeste homolog 2, and *SMARCA4*. Among these genes, *HDAC1* and *HDAC2* are targets of TSA, which is a potent inhibitor of *HDACs* [37,58]. In M17 and M18, *HDAC1* and *HDAC2* linked to other genes to perform different functions. For example, *DNMT1* in module M17 can be translated into a homodimer and form a stable complex with *HDAC1* protein, inhibiting the transcription of the E2F-responsive promoter [59]. The combination of *HDAC2*, *HDAC1*, and *RBBP4* genes in module M18 can form part of the core *HDAC* complex, and TSA chelates zinc ions in the recesses of the active site through the hydroxamic acid group of *HDAC*. In turn, this interaction prevents the catalytic action of *HDAC* [60]. The transcriptional translation of *SMARCA4* in modules M17 and M18 is followed by part of the *CREST-BRG1* complex, and the activity-dependent induction of N-methyl d-aspartate receptor subtype 2B (*NR2B*) expression involves the release of the *HDAC1* gene. However, the *CREST-BRG1* complex binds to the *NR2B* promoter and participates in transcriptional activation and selection of the gene’s inhibitory process, thereby affecting the release of the *HDAC1* gene [61].

We further performed literature analysis to verify the relationship of the genes in M17 and M18 with TSA. *SMARCA4* (also known as *BRG1*) has been shown to be involved in various developmental processes, transcriptional regulation, DNA repair, cell cycle regulation, and cancer [62]. *SMARCA4* is an ATPase subunit essential for the *SWI/SNF* chromatin remodeling complex in mammals and is also involved in expression of the tumor-suppressor gene *SNF2β* [63]. Moreover, *SMARCA4* can destroy the target region’s nucleosomes by using the energy generated by ATPase hydrolysis [63]. Mackmull et al. [64] found that *SMARCA4* is affected at the protein level after 12h of TSA treatment, and its abundance at the protein level is regulated by a combination of transcriptional and post-transcriptional mechanisms. Using gene silencing techniques, they demonstrated that a decrease in *SMARCA4* richness is sufficient to regulate transcriptional changes induced by TSA [64].

*PARP1* modifies various nuclear proteins by encoding a chromatin-associated poly ADP-ribosyltransferase, which is dependent on DNA and participates in a variety of important cellular processes that regulate molecular activity associated with cellular recovery from DNA damage [65]. Nonhomologous end joining (*NHEJ*) is one of the major mechanisms through which DNA double-strand breaks (*DSBs*) are repaired. TSA significantly increases the probability of *PARP1* binding to chromatin *DSBs* and the likelihood that poly ADP-ribose will colocalized with *DSBs* in TSA-treated patients with leukemia [62]. In addition, knockout of *PARP1* inhibits the effects of TSA on *NHEJ*. Thus, these results indicated that administration of TSA can reduce the cytotoxicity of *NHEJ* in leukemia cells [66].

## 4. Conclusions

In biological networks, a drug’s therapeutic action disturbs the network. Drugs typically show therapeutic activity in many diseases. Thus, drug therapy exhibits certain types of patterns, such as unique modules related to the drug. In this report, we attempted to identify the treatment patterns, i.e., target modules in multilayer networks, of TSA using a new framework. Drugs affect different diseases by acting on gene sets with similar network structures. Because diseases are typically tissue specific, there are common and distinct relationships among the same gene sets in different protein-protein interaction networks. Therefore, we used TSA as an example to establish a three-layer TSPPI network with a tensor-based multilayer network mining algorithm [43] to identify the TSA treatment pattern. Using the multilayer network mining algorithm, it was possible to extract the structure of the same gene set with different connections in different layers. Finally, we identified modules M17 and M18 as potential treatment patterns for TSA. We verified the results from multiple perspectives, including difference and functional enrichment comparisons, co-expression network analysis, and literature verification. For example, if a disease-related protein module has a similar structure as M17 or M18, then the drug TSA is likely to be a potential drug for the treatment of the disease.

There are still some limitations to our method. First, the TSPPI networks were incomplete and had false-positive connections. Second, despite our extensive analysis, more studies are required for biological validation of the data. In future studies, we will attempt to overcome these shortcomings and further improve the framework to make the results more reliable.

## Figures and Tables

**Table 1:** Selected candidate drug-target modules based on genes affected by TSA.

Module ID	Entrez IDs of Genes in the Modules
M1	890, 7153, 4085, 6241, 701, 22974, 6790, 3161, 11130, 10403, 6240, 10051, 51203, 1434, 1719, 3832, 7298, 5984, 10592, 4173, 891, 9319, 2237, 3838, 990, 47, 90, 87
M2	7520, 142, 1019, 5111, 5591, 6749, 2237, 5036, 4522, 6241, 4175, 10606, 5982, 1736
M3	22948, 10213, 10969, 471, 1434, 3329, 5686, 1503, 9221, 908, 5901, 5036, 3838, 7371
M4	5901, 7334, 7520, 7443, 10576, 7153, 10213, 26135, 6636, 6427, 5902, 6428, 6240
M5	22948, 7203, 6950, 10574, 11222, 1164, 4830, 7334
M6	4172, 6627, 1503, 10528, 11130, 2237, 7398, 9521, 5985
M7	6426, 4436, 10772, 10236, 3838, 26135, 1665, 23165, 10576, 7520
M8	7153, 5557, 6790, 672, 8317, 10733, 4001, 1736
M9	6426, 9221, 6434, 7334, 3015, 1736, 2237, 3184, 2956, 6427
M10	10574, 158, 7965, 142, 1503, 7411, 4176, 1736, 8607, 7203, 5901, 5902
M11	6637, 5111, 3148, 3182, 6434
M12	1434, 3308, 908, 4869, 6950, 7203, 3336, 3838
M13	10492, 1503, 3182
M14	3276, 5725, 3609, 6597, 4176, 6627
M15	6194, 6124, 6201, 6137, 11224, 6143, 6193, 6217, 6152, 6139, 6136, 6161, 23521, 6133, 6175, 4736, 6207, 6218, 6135, 6128, 6146, 3646, 1933, 47, 87, 39, 29, 90, 95
M16	3014, 84823, 6597, 5036
M17	3065, 142, 1786, 6597
M18	3066, 3065, 5928, 2146, 6597
M19	86, 6597, 10856
M20	6597, 6599, 5591, 4173, 4172
M21	6597, 23246, 8662
M22	5036, 10574, 3182
M23	3329, 7203, 6428
M24	10606, 6950, 4691, 3183, 6741, 3843, 5901
M25	890, 7371, 3251, 1665
M26	5557, 990, 9493, 9833, 1060

**Table 2:** KEGG pathway enrichment of conserved first-order genes of modules in the blood TSPPI network.

Module	Threshold of Score	Total Number of Genes	Number of KEGG Pathways
M2	7.0	46	12
M17	2.0	21	11
M18	2.1	18	6
M20	2.3	35	4

**Table 3:** KEGG pathway enrichment of conserved first-order genes of modules in the breast TSPPI network.

Module	Threshold of Score	Number of Total Genes	Number of KEGG Pathways
M2	7.2	59	13
M17	2.0	29	10
M18	2.0	28	6
M20	2.5	42	5

**Table 4:** KEGG pathway enrichment of conserved first-order genes of modules in the prostate TSPPI network.

Module	Threshold of Score	Number of Total Genes	Number of KEGG Pathways
M2	4.5	65	12
M17	1.2	28	15
M18	1.4	21	14
M20	1.8	30	5

**Table 5:** The number of KEGG pathways that overlapped between the first-order genes of the module and the TSA targets in three TSPPI networks.

Tissue	M2	M17	M18	M20
Blood	0	3	1	0
Breast	1	4	1	0
Prostate	1	4	4	1

**Table 6:** The number of overlapping KEGG pathways enriched by first-order genes of M17 and M18 and disease-causing genes for leukemia, breast cancer, and prostate cancer.

Cancer	M17	M18
Leukemia	6	1
Breast cancer	3	1
Prostate cancer	1	1

**Table 7:** Significance of target modules M17 and M18 for TSA in the three TSPPI networks.

Tissue	p Value for M17	p Value for M18
Blood	6.27 × 10^4^	6.36 × 10^6^
Breast	3.24 × 10^4^	0
Prostate	1.64 × 10^4^	0

**Table 8:** Significance of target modules for TSA in five other TSA-related PPI networks.

Tissue	Number of Edges	Minimal Edge Weight	p Value for M17	p Value for M18	
Lung	149,495	0.374935	2.12 × 10^4^		0
Colon	163,180	0.317351	6.91 × 10^4^		0
Ovarian	161,487	0.37902	2.67 × 10^4^		0
Pancreas	161,147	0.312249	6.58 × 10^4^		8.43 × 10^5^
Marrow	154,621	0.391356	0.0242		9.23 × 10^4^
